# The Aix Douche-Massage

**Published:** 1900-05-19

**Authors:** 


### THE AIX DOUCHE-MASSAGE.

Aix-les-Bains is not only a popular and fashionable watering-place, but one which has developed a special method for the treatment of certain diseases of the joints—the douche-massage or massage under the constantly flowing douche, which is well described by Dr. Forestier.[1] This general douche-massage is performed by two masseurs or masseuses as the case may be. The patient, if crippled as in rheumatoid arthritis, is brought in a *chaise à porteurs* or sedan chair wrapped up in two or three woollen blankets. The massage is performed in two stages, which are in some cases supplemented by other proceedings. The first part of the douche-massage is performed in the sitting posture, the patient sitting on a wooden stool, while both the masseurs (or masseuses) do the massage of the limbs and of the upper part of the body, all the time pouring the jet of warm water on to the part they are manipulating. The attendants are very skilful in directing the jet by the raised knee, so that both their hands are at liberty for the massage. One masseur standing behind the patient does the massage of the neck, shoulders, and upper part of the trunk, while the other standing in front massages the upper and lower limbs. The second part of the douche-massage is done with the patient in a reclining posture, lying on a wooden board put on the stool so as to make an inclined plane. While in that position it is very easy for the masseur to massage the lumbar region, the hip-joint and surrounding parts, and the sciatic nerve running along the posterior aspect of the limb, all of which require some skill and force. So much for the actual massage, the Aix-douche as it is sometimes called. After this is over the patient stands up, and while one of the attendants prepares the linen the other gives a simple douche more or less warm for one or two minutes, and in some cases in which a tonic effect is desired this final douche is warm and cold, when it is called the Scottish douche. As to the temperature of the water in the douche-massage it may be from 90 deg. F. to 104 deg. F., or, exceptionally, to 107 deg. F.; and as to the sort of massage used, it is prescribed by the doctor, and may take the form of effleurage, pétrissage, or pressure, &c. Its duration is ten minutes. The accessory treatment which is employed in some cases may be either (1) the general vapour bath, known as Bouillon from the bubbling noise made by the shower of hot water from which the vapour is given off. In this chamber patients may stay from three to ten minutes before or after the douche-massage, so as to get an abundant perspiration. (2) The local vapour bath, known as Berthollet, which lasts from 20 to 30 minutes, and is generally followed by a dry massage, the temperature being 100 deg. F. to 110 deg. F. (3) The ordinary tub bath. As a rule, a course of about twenty douche-massages is required, spread over about a month; but in severe cases the course lasts longer, and the tub and vapour baths are used as well as the more special douche-massage.

[1] Practitioner, May, 1900.

